# SARS-CoV-2 SPIKE PROTEIN: an optimal immunological target for vaccines

**DOI:** 10.1186/s12967-020-02392-y

**Published:** 2020-06-03

**Authors:** Giovanni Salvatori, Laura Luberto, Mariano Maffei, Luigi Aurisicchio, Giuseppe Roscilli, Fabio Palombo, Emanuele Marra

**Affiliations:** 1 Takis s.r.l, Via di Castel Romano 100, 00128 Rome, Italy; 2 Evvivax s.r.l, Via di Castel Romano, 100, 00128 Rome, Italy

**Keywords:** SARS-CoV-2, SPIKE protein, Vaccine

## Abstract

COVID-19 has rapidly spread all over the world, progressing into a pandemic. This situation has urgently impelled many companies and public research institutes to concentrate their efforts on research for effective therapeutics. Here, we outline the strategies and targets currently adopted in developing a vaccine against SARS-CoV-2. Based on previous evidence and experience with SARS and MERS, the primary focus has been the Spike protein, considered as the ideal target for COVID-19 immunotherapies.

## Main text

The outbreak of the novel coronavirus disease COVID-19, now officially designated as severe acute respiratory syndrome-related coronavirus SARS-CoV-2, has progressed rapidly into a pandemic. In just a few months, since December 2019, COVID-19 has spread worldwide with over 4.218.212 confirmed cases and more than 290.242 confirmed deaths as of May 14th, 2020 (WHO, Situation).

This dramatic situation calls for the rapid development of safe and effective prophylactics and therapeutics against infection of its causative agent. To date, no therapeutics or vaccines against any human-infecting coronaviruses have been approved.

Currently, ongoing strategies to trigger an effective immune response in humans against SARS-CoV-2 are taking advantage of previous experiences on other coronaviruses such as SARS-CoV and MERS-CoV. Since the SARS-CoV-2 virus shares striking structural similarity and sequence conservation with these two lethal coronaviruses, the immunization strategies exploited against SARS and MERS viruses have been adopted in guiding the design of new SARS-CoV-2 vaccines.

Immunization with one or more SARS-CoV-2 subunit antigens, either administered as purified protein or expressed by viral, RNA or DNA vaccine vectors, is one approach to designing a vaccine.

Among the more likely targets for vaccination are the structural proteins that bedeck the surface of SARS-CoV-2. These include the envelope spike protein S, the small envelope protein E, the matrix protein M and the unexposed nucleocapsid protein N.

An early study on recombinant vectors expressing the S protein of SARS-CoV found this protein to be highly immunogenic and protective against SARS-CoV challenge in hamster, while in contrast, the N, M, and E proteins did not significantly contribute to a neutralizing antibody response or protective immunity [1].

Evidence of the key role played by the S protein in counteracting coronavirus infection came from studies on human-neutralizing antibodies from rare memory B cells of individuals infected with SARS-CoV [2] or MERS-CoV [3]. In such studies, antibodies directed against the S protein of SARS-CoV were found effective in inhibiting virus entry into the host cells. More recently, it has been found that SARS-CoV S elicited polyclonal antibody responses, and vigorously neutralized SARS-CoV-2 S-mediated entry into cells, thus further encouraging the use of this molecular target for vaccination and immunotherapies [4].

Structural studies of antibodies in complex with SARS-CoV S and MERS-CoV S have provided information about the mechanism of competitive inhibition to the host receptor. The receptor-binding domain (RBD) in SARS-CoV-2 S protein was identified and found to bind strongly to ACE2 receptors [5]. SARS-CoV RBD-specific antibodies cross-react with SARS-CoV-2 RBD protein, and SARS-CoV RBD-induced antisera neutralized SARS-CoV-2, providing additional evidence that targeting this domain of the S protein of SARS-CoV-2 with a vaccine could be effective in preventive COVID-19 [5].

Given the above and that the coronavirus S glycoprotein is surface-exposed and mediates entry into host cells by interacting with angiotensin-converting enzyme 2 (ACE2), it rapidly became the main target of neutralizing antibodies and the focus of therapeutic and vaccine design.

Several companies and research institutes have started developing a vaccine that has the SARS-CoV-2 protein S as its target (see Table 1), although the various vaccination strategies show a differing ability to induce in the host both an antibody-mediated humoral response and a cell response mediated by CD4 or CD8 T lymphocytes in preclinical models.

**Table 1:** Developmental vaccines targeting SARS-CoV-2 protein S (Adapted from BioWorld, company sites, Thomsen Cortellis, PubMed)

Companies	Vaccination typology	Current development stage
Altimmune	A replication-defective adenovirus vector incorporating the SARS-CoV-2 S protein administered by an intranasal single-dose	The vaccine design and synthesis steps are completed. Moving toward preclinical tests and manufacture, hoping to start phase 1 trial at mid-August
CanSino biologicals	Adenovirus type 5 vector that expresses S protein	Phase I (NCT04313127) completed Phase II started: It is China’s first recombinant vaccine candidate for novel coronavirus entering Phase II of a human clinical trial, with 500 volunteer participants
(Sichuan) Clover Biopharmaceuticals (Chengdu, China) Partnered with GlaxoSmithKline	Recombinant SARS-CoV-2 S-protein trimer subunit produced by its patented Trimer-Tag© technology	Carrying out preclinical tests with GlaxoSmithKline’s pandemic vaccine adjuvant technology and in collaboration with Dynavax, proprietary holder of toll-like receptor 9 agonist adjuvant, CpG 1018
Inovio Pharmaceuticals	Electroporation of DNA INO-4800 encoding SARS-CoV-2 S protein	Started trial in United States (Phase 1 NCT04336410). There are already 3000 doses available
LineaRx Takis Biotech (Rome) to clinical test candidates in Italy	Electroporation of linear DNA encoding S protein or its specific portions	Five candidates have been designed of linear DNA vaccine based on S protein and selected epitopes, ready for testing by the beginning of May or June
Moderna	The mRNA encoding SARS-CoV-2 S protein is encapsulated in ionizable lipid, distearoyl phosphatidylcholine, cholesterol and polyethylene glycol lipid	Phase 1 (NCT04283461) testing is underway
Novavax	Nanoparticle displaying SARS-CoV 2 S protein with saponin-based (Matrix-M) adjuvant	Currently assessing the candidates in animal models, expecting to start Phase 1 trial in June 2020
University of Queensland (Brisbane, Australia)	Recombinant subunit of SARS-CoV-2 S protein locked in prefusion conformation by polypeptide moiety (molecular clamp)	In preclinical testing, partnering with Dynavax Technologies Corp. in collaboration with GlaxoSmithKline plc and Seqirus GmbH

The evolving molecular heterogeneity of SARS-CoV has raised concerns about the breadth and efficacy of protection provided by specific vaccine strains and the possible development of immune escape. However, it has been observed that a heterotypical response blocking SARS-CoV-2 S-mediated entry into host cells is elicited, coinciding with the sequence and structural conservation of SARS-CoV-2 and SARS-CoV S protein, suggesting that immunity against one virus can potentially provide protection against related viruses.

One of the most perplexing questions regarding the current COVID-19 coronavirus epidemic is the possible worsening of the disease by immunotherapies, as the consequence of an antibody dependent enhancement (ADE) of infection with SARS-CoV-2. ADE of viral entry has been a major concern for epidemiology, vaccine development, and antibody-based drug therapy. ADE viral entry into the target cell of SARS-CoV-2 is mediated by the Fc receptor II and not by its canonical receptor. It has been suggested that ADE may explain geographical differences in the severity of COVID-19 due to prior exposure to similar antigenic epitopes [6].

One study showed that the antibody against SARS-CoV spike protein potentiated infection of monocytes. However, ADE-infected macrophage did not support the productive replication of SARS-CoV, and no detectable release of progeny virus was observed [7]. In a mouse model of vaccination for SARS-CoV with different approaches including inactivated virus, DNA or recombinant spike (S) protein, vaccines lead to pulmonary immunopathology. However, despite deterioration in the pulmonary histopathology profile of the vaccinated mice, all the SARS-CoV vaccines induced antibody and protection against infection with SARS-CoV [8].

It has been found that higher concentrations of anti-sera against SARS-CoV neutralized infection, while highly diluted anti-sera significantly increased SARS-CoV infection. Results from infectivity assays indicate that SARS-CoV ADE is primarily mediated by diluted antibodies against envelope spike protein [9]. However, the relevance of ADE in coronavirus infections remains elusive, as no direct evidence of it has been found in various vaccination models [5]. Accordingly, it has been shown that vaccination of the Rhesus Macaque monkey with an attenuated SARS-CoV revealed no exacerbation of infection even several weeks after vaccination, when the antibody titer was reduced [10].

It is of relevance that several companies are involved in the development of a vaccine against the spike protein of SARS-CoV-2, exploiting different strategies such as purified protein or expressed by viral, RNA or DNA vaccine vectors. This target has been guided by previous preclinical history of the proven efficacy of immunotherapies against the homologous protein of SARS-CoV. Although the ADE effect of non-neutralizing antibodies directed against the SARS-CoV-2 S protein remains controversial, safety testing of COVID-19 S protein-based B cell vaccines in animal models is strongly encouraging prior to clinical trials. Given the urgency of an effective vaccination to prevent the spread of SARS-CoV-2, this plurality of approaches in vaccine generation with complementary strategies, paves the way to a wider immunotherapeutic spectrum, thus increasing the chances of success in such a short time frame.

## Data Availability

Not applicable.

## Abbreviations

ACE2: Angiotensin-converting enzyme 2
RBD: Receptor-binding domain
ADE: Antibody dependent enhancement
